# Immune reconstitution 20 years after treatment with alemtuzumab in a rheumatoid arthritis cohort: implications for lymphocyte depleting therapies

**DOI:** 10.1186/s13075-016-1188-6

**Published:** 2016-12-20

**Authors:** Faye A. H. Cooles, Amy E. Anderson, Tracey Drayton, Rachel A. Harry, Julie Diboll, Lee Munro, Nishanthi Thalayasingham, Andrew J. K. Östör, John D. Isaacs

**Affiliations:** 1 Institute of Cellular Medicine, Newcastle University and National Institute for Health Research Newcastle Biomedical Research Centre at Newcastle upon Tyne Hospitals NHS Foundation Trust and Newcastle University, Newcastle upon Tyne, UK; 2 Addenbrooke’s NHS trust, Cambridge, UK

**Keywords:** Lymphodepletion, Alemtuzumab, Rheumatoid arthritis, Immune homeostasis, CD5^+^ B cells

## Abstract

**Background:**

Alemtuzumab, an anti-CD52 monoclonal antibody, was administered to patients with RA between 1991 and 1994. We have followed a cohort of recipients since that time and previously reported significant delays in immune reconstitution. Here we report >20 years of follow-up data from this unique cohort.

**Method:**

Surviving alemtuzumab recipients were age, sex and disease duration matched with RA controls. Updated mortality and morbidity data were collected for alemtuzumab recipients. For both groups antigenic responses were assessed following influenza, Pneumovax II and combined diphtheria/tetanus/poliovirus vaccines. Circulating cytokines and lymphocyte subsets were also quantified.

**Results:**

Of 16 surviving alemtuzumab recipients, 13 were recruited: 9 recipients underwent a full clinical assessment and 4 had case notes review only. Since our last review 10 patients had died from causes of death consistent with long-standing RA, and no suggestion of compromised immune function. Compared with controls the alemtuzumab cohort had significantly reduced CD4^+^ and CD8^+^ central memory T-cells, CD5^+^ B cells, naïve B cells and CD19^+^CD24^hi^CD38^hi^ transitional (putative regulatory) B cells. Nonetheless vaccine responses were comparable between groups. There were significantly higher serum IL-15 and IFN-γ levels in the alemtuzumab cohort. IL-15 levels were inversely associated with CD4^+^ total memory and central memory T cells.

**Conclusion:**

After 20 years the immune system of alemtuzumab recipients continues to show differences from disease controls. Nonetheless mortality and morbidity data, alongside vaccination responses, do not suggest clinical immune compromise. As lymphodepleting therapies, including alemtuzumab, continue to be administered this work is important with regard to long-term immune monitoring and stages of immune recovery.

## Background

Targeted immunomodulatory therapies have revolutionised the treatment of autoimmune diseases, particularly rheumatoid arthritis (RA). Alemtuzumab (CAMPATH-1H) was the first humanised therapeutic monoclonal antibody and acts by depleting cells expressing CD52, which includes T and B lymphocytes, monocytes, and natural killer (NK) cells. Alemtuzumab was initially used with some success to treat several cohorts of refractory RA patients in the early 1990s; however, given the resultant profound lymphopenia, particularly in the CD4^+^ compartment, trials in RA were curtailed [1]. We previously reported delayed reconstitution of the immune system following the treatment of RA with alemtuzumab, particularly of T cells and CD5^+^ B cells, but with no apparent adverse effects on 12-year morbidity or mortality or on the response to antigenic challenges [2, 3].

Iatrogenic lymphopenia is becoming increasingly relevant as depleting therapies are more widely used in the treatment of cancer, transplantation and also autoimmunity. For example alemtuzumab is now licenced for use in relapsing-remitting multiple sclerosis [4]. Furthermore there are suggestions that rituximab, an increasingly commonly administered B cell depleting therapy for the treatment of RA, also depletes CD4^+^ T cells [5]. Thus, understanding the dynamics of immune reconstitution and the long-term outcome of depleting therapies is becoming increasingly important in wider clinical practice. We report here the 20-year follow up of our original cohort of patients with RA treated with alemtuzumab.

## Methods

### Patients and controls

Patients who were treated with alemtuzumab (CAMPATH-1H) for RA between 1991 and 1994 were identified from the study database. Cumulative alemtuzumab doses were documented for each individual; of note, these doses were comparatively lower than those seen with other more widely used biological therapies. Mortality data for this cohort were collected from 9 March 2006 (end date of our previous study) until 1 January 2015 from the National Health Service Central Registry. Morbidity information was collected on all living and consenting patients by either interview or review of clinical case notes. Special attention was given to episodes of severe infection, cancers and autoimmunity. Age and sex matched patients with RA of similar disease duration, who had not received alemtuzumab, were identified locally in Cambridge, UK. Research was performed in compliance with the Declaration of Helsinki and the International Conference on Harmonisation Good Clinical Practice. Ethical approval for the study was provided by Scotland A Research Ethics Committee (REC 10/MRE00/68).

### Clinical parameters

Clinical and serological parameters were obtained from all patients attending interview. Markers of disease activity included erythrocyte sedimentation rate (ESR), C-reactive protein (CRP), tender joint count (TJC), swollen joint count (SJC), patient-reported visual analogue scale (VAS), disease activity score in 28 joints (DAS-28)-ESR and patient health assessment questionnaire (HAQ). Anti-cyclic citrullinated peptide (anti-CCP) antibody and rheumatoid factor (RF) IgM titres and serum immunoglobulins (IgG, IgA, IgM), serum electrophoresis and lymphocyte count were analysed in the clinical laboratories of Addenbrooke’s Hospital, Cambridge, according to routine clinical practice and national standards.

### Vaccine responses

Subjects were offered vaccination with: 0.5 ml influenza vaccine (Pfizer Ltd, Sandwich, UK), a split, inactivated influenza vaccine containing antigens equivalent to A/California/7/2009 (H1N1) pdm09-derived strain (NYMC X-181), A/Victoria/361/2011 (H3N2)-derived strain (IVR-165) and B/Wisconsin/1/2010–like strain (NYMC BX-39) derived from B/Hubei- B/ Wujiagang/158/2009; 0.5 ml Pneumovax II (Sanofi Pasteur MSD Ltd, Maidenhead, UK), a vaccine containing 25 μg of each of the following 23 pneumococcal polysaccharide serotypes: 1, 2, 3, 4, 5, 6B, 7 F, 8, 9 N, 9 V, 10A, 11A, 12 F, 14, 15B, 17 F, 18C, 19 F, 19A, 20, 22 F, 23 F, 33 F; and 0.5 ml Revaxis (Sanofi Pasteur MSD Ltd, Maidenhead, UK) containing 2 IU purified diphtheria toxoid, 20 IU purified tetanus toxoid and inactivated poliomyelitis virus types 1-3.

Vaccine responses were assessed in serum obtained at baseline and 4 weeks post vaccination. Analyses were performed at the Respiratory Virus Unit, Health Protection Agency, London UK (influenza), the Vaccine Evaluation Unit, Public Health England, Manchester (pneumococcus, diphtheria and tetanus) and Public Heath England, Enteric Virus Unit, London (poliovirus). Satisfactory response to pneumococcal vaccine was defined as a doubling (or greater) in antibody concentrations to 6 or more of 12 pneumococcal serotypes (1, 3, 4, 5, 6B, 7 F, 9 V, 14, 19A, 19 F, 23 F and 18C). Tetanus and diphtheria seroprotection was achieved when IgG titres were >1.0 IU/ml. These were booster vaccines and some patients had residual seroprotection pre-administration. These patients were excluded from subsequent seroconversion analysis, which was defined as when vaccination achieved new seroprotection.

For poliovirus, neutralizing antibodies were quantified, with seroprotection with titres ≥1:8 and seroconversion following ≥4 fold increase in titres. For influenza, HAI assays were performed, with seroprotection when titres were >1:40 and seroconversion when post-vaccination titre increased by ≥3 fold. The seroconversion factor was the mean rise in geometric mean titres (GMT) post vaccination (recommended ≥2) and seroconversion rate was the percentage of vaccinees with an increase in haemagglutination inhibition (HAI) titre ≥4 fold following vaccination (recommended >30%). Some patients had annual influenza vaccines prior to recruitment into the study. These individuals were not vaccinated again but anti-influenza titres were measured in baseline serum. Any adverse effects were collected 4 weeks post vaccination.

### Serum cytokines

At the initial visit cytokines (IL-15, IL-7, IFN-γ, IL-10, IL-12, IL-13, IL1-beta, IL-2, IL-4, IL-6, IL-8, TNF-alpha and granulocyte macrophage colony stimulating factor (GM-CSF)) were measured in serum by MSD technology (Meso Scale Discovery, MD, USA) as per established protocol.

### Immunophenotyping

Peripheral blood lymphocytes were immunophenotyped by multicolour flow cytometry using the following antibodies: anti-CD3 Pacific Blue, anti-CD56 FITC, Anti-CD27 PE, anti-CD28 APC, anti-CD1d PE, anti-CD19 APC, anti-CD27 V450, anti-CD38 PerCP-Cy5.5 (all from BD Biosciences, San Jose, CA, USA) and anti-CD45RA PerCP-Cy5.5, anti-CD62L PE-Cy7, anti-CD5 PE-Cy7, anti-CD24 APC-eFluor 780 and anti-CD4 APC-eFluor 780 (from eBioscience, Inc. San Diego, CA, USA). Staining was performed on whole blood using BD FACS Lysing Solution (BD Biosciences) as per the manufacturer’s instructions. A minimum of 250,000 events were acquired for T cell panels and 500,000 events for B cell panels to ensure adequate capture of rare populations. Subsequent detailed analysis of lymphocyte sub-populations was performed on the gated lymphocyte population using FlowJo (Treestar, Inc., OR, USA). Absolute counts for the different lymphocyte populations were calculated per litre of blood, based on haematology laboratory reported total lymphocyte count.

### Statistical analysis

Statistical analysis was performed using the Mann-Whitney *U* test, Wilcoxon signed rank test and linear regression using Prism 4.0 (GraphPad Software, Inc., La Jolla, CA, USA). *P* values <0.05 were considered significant.

## Results

### Demographics

Sixteen patients from the original alemtuzumab cohort were alive at the time of recruitment. Nine agreed to be interviewed and to provide a blood sample in conjunction with vaccination. An additional four patients agreed to their clinical notes being reviewed, two declined and one could not be contacted. A further eight age and sex matched patients with established RA of a similar disease duration were recruited as controls. Cohort demographics, current treatment and (where applicable) past alemtzumab treatment dose are shown in Tables 1, 2 and 3.

**Table 1:** Alemtuzumab patient and RA patient control demographic and serological data. CAM prefix denotes alemtuzumab treated patients and CON prefix denotes control patients. ^a^Total cumulative alemtuzumab dose administerd to RA patients between 1991–1994. ^b^DAS-28-ESR at the baseline visit for this study; ^c^VAS pain patient reported; ^d^SJC: swollen joint count, range 0–28; ^e^TJC: tender joint count, range 0–28; ^f^IgG: normal range: 6-16 g/L; ^g^IgA: normal range: 0.9-4.5 g/L; ^h^IgM normal range: 0.5-2 g/L; ^i^RF titre by direct quantification, IU/ml; positive threshold 14 IU/ml; ^j^CCP: anti-cyclic citrullinated peptide, U/ml, positive threshold 7 U/ml; ^k^DMARD/Biological therapy: MTX - methotrexate; HCQ – hydroxychloroquine; LEF–leflunomide; SSZ-sulphasalazine; ABA–abatacept; ADA – adalimumab; ETA – etanercept; TOC – tocilizumab; n/a: not applicable

	Age (Years)	Sex	Disease Duration (Years)	Alemtuzumab total dose (mg)^a^	DAS-28^b^	CRP (mg/L)	ESR (mm/hr)	HAQ	VAS^c^	SJC^d^	TJC^e^	IgG^f^ (g/L)	IgA^g^ (g/L)	IgM^h^ (g/L)	RF^i^ (IU)	CCP^j^ (U/ml)	DMARD Therapy^k^
CAM05	55	F	36	250	4.27	4	26	1.625	56	2	6	12.2	2.9	0.06	0	0	AZA, ETA
CAM10	65	M	38	18	3.36	26	34	2.000	25	0	1	10.7	2.3	0.60	0	1.4	LEF, MTX
CAM21	45	F	23	18	1.38	2	4	2.250	18	2	6	n/a	n/a	n/a	0	0.5	LEF, MTX
CAM26	69	F	26	30	4.74	7	14	2.250	49	0	0	6.2	1.1	0.70	25	0	HCQ, LEF
CAM27	56	M	28	400	2.20	7	15	2.000	8	0	0	8.0	3.2	3.20	0	0.6	MTX
CAM29	81	F	24	30	3.44	7	52	2.250	4	0	0	8.3	1.9	1.00	67	n/a	MTX
CAM32	74	F	31	184	4.55	12	104	2.125	36	0	0	16.6	4.2	1.40	20	340	ETA
CAM46	68	F	40	250	5.33	134	73	2.750	40	5	6	11.4	3.6	1.40	0	0	ABA
CAM52	55	F	34	60	2.90	22	40	3.000	2	1	1	7.4	1.1	0.70	0	0.7	LEF, MTX
CON1	54	F	41	n/a	5.75	2	8	1.125	75	7	20	11.4	3.9	0.80	0	n/a	SSZ, HCQ
CON2	52	F	32	n/a	4.83	1	24	2.000	22	5	3	4.8	2.0	0.30	39	n/a	AZA
CON3	65	F	20	n/a	2.04	1	2	1.375	14	0	1	4.4	0.4	0.90	179	152	TOC, SSZ
CON4	76	F	22	n/a	2.27	2	2	1.625	11	1	3	10.3	1.8	0.70	33	294	TOC, MTX
CON5	79	F	21	n/a	2.97	1	14	2.250	24	0	1	11.8	2.7	0.60	137	215	ABA, MTX
CON6	67	F	33	n/a	5.12	1	38	1.750	39	5	7	16.3	2.8	2.50	16	n/a	ETA
CON7	54	F	20	n/a	2.03	2	14	0.250	4	0	0	9.3	1.2	1.30	0	1.2	MTX, SSZ, HCQ
CON8	58	M	27	n/a	5.11	1	9	2.125	55	1	17	9.6	1.8	1.00	275	340	MTX

**Table 2:** Alemtuzumab patient and RA patient control pooled demographic and serological data. *P* value: alemtuzumab patients vs established controls. Values in italics are significant (*p* < 0.05)

Characteristic	Alemtuzumab Treated patients	RA Control patients	*P* value
Age (Years) median, range	65 [45–81]	61.5 [52–79]	0.773
Sex male/female, %	77% F	87.5% F	0.600
Disease Duration (Years) median, range	31 [23–40]	24.5 [20–41]	0.200
Alemtuzumab total dose (mg) median, range	60 [18–400]	-	-
DAS-28 median, range	3.44 [1.38-5.33]	3.9 [2.03-5.75]	0.743
CRP (mg/L) median, range	7 [<5-134]	<5 [all <5]	*0.001*
ESR (mm/hr) median, range	34 [4–104]	11.5 [2–38]	*0.03*
HAQ median, range	2.25 [2–3]	1.69 [0.25-2.13]	*0.025*
VAS median, range	25 [2–56]	23 [4–75]	0.847
SJC (n) median, range	0 [0–2]	1 [0–7]	0.414
TJC (n) median, range	1 [0–6]	3 [0–20]	0.154
IgG (g/L) median, range	9.5 [6.2 – 16.6]	9.95 [4.4-16.3]	0.916
IgA (g/L) median, range	2.6 [1.1 – 4.2]	1.9 [40.4 -3.9]	0.431
IgM (g/L) median, range	0.85 [0.006 - 3.2]	0.85 [0.3 -2.5]	0.874
RF (IU) median, range	0 [0–67]	39 [0–275]	0.060
CCP (U/ml) median, range	0.55 [0–340]	215 [1.2-340]	*0.039*

**Table 3:** Alemtuzumab patient demographical data for whom only morbidity and current conventional synthetic or biological DMARD therapy data was obtained

	Age (Years)	Sex	Disease Duration (Years)	Alemtuzumab total dose (mg)	DMARD/Biological therapy
CAM14	68	F	35	62	MTX
CAM15	69	M	23	400	HCQ
CAM16	75	F	26	260	MTX, HCQ
CAM24	63	M	43	2	ADA

MTX - methotrexate; HCQ – hydroxychloroquine; ADA – adalimumab

Briefly, the median age of the entire alemtuzumab cohort was 68 (range 45–81) years, 69% were female, the median disease duration was 31 years (range 23–43) and the median alemtuzumab cumulative dose was 62 mg (range 2–400 mg). Patients who underwent vaccination and peripheral blood analysis had a median age of 65 years (range 45–81), 77% of them were female, the median disease duration was 31 years (range 23–40), the median DAS-28 was 3.44 (range 1.38–5.33) and the median alemtuzumab cumulative dose was 60 mg (range 18–400 mg). The median age in the controls was 61.5 years (range 52–79), 87.5% were female, the median disease duration was 24.5 years (range 20–41) and the median DAS-28 was 3.90 (range 2.03–5.75). There was no significant difference in age, sex, disease duration, SJC, TJC or DAS-28 between the cohorts that underwent vaccination. The alemtuzumab cohort had a higher HAQ score (median 2.25 compared with 1.69 in controls), CRP (median 7 compared with <5 in controls) and ESR (median 34 compared with 11.5 in controls).

Nine of the alemtuzumab cohort were taking conventional synthetic disease-modifying anti-rheumatic drugs (csDMARDs) only, three were receiving biological DMARD (bDMARD) monotherapy and one was receiving concurrent bDMARDs and csDMARDs. One of these patients had recently received rituximab prior to being switched to their current therapy (abatacept) and was therefore excluded from B cell analysis. No further patients in either the control or alemtuzumab cohorts had previously received rituximab. In the control cohort four patients were taking csDMARDs only, three were receiving both csDMARDs and bDMARDs and one was receiving bDMARD monotherapy.

There was no significant difference in immunoglobulin titres (IgG, IgA and IgM) between the alemtuzumab and established RA control cohort. RF titres were performed in all patients, and anti-CCP antibody titres were obtained in eight patients taking alemtuzumab and five controls. The median RF titre inr the alemtuzumab cohort was 0 IU, (range 0–67 IU) and in the controls the median was 39 IU (range 0–275) (*p* = 0.06). The median anti-CCP value in alemtuzumab patients was 0.55 U/ml (range 0–340) and in controls it was 215 U/ml (range 1.2–340) (*p* = 0.039).

### Mortality and morbidity

Ten patients in the alemtuzumab cohort had died since our previous review. Causes of mortality, as per death certificate documentation, are outlined in Table 4. Three cases of malignancy were noted (brain tumour, lung adenocarcinoma and carcinomatosis with unknown primary). Infection was listed as cause of/contributor to death in four patients (three patients with respiratory (one with concurrent cellulitis) and one with urinary origin). Cardiovascular disease was a contributor in two patients (congestive cardiac failure and vascular dementia) and was implicated in one other (respiratory failure and congestive cardiac failure). The remaining causes/contributors to death were dementia, pulmonary fibrosis and chronic obstructive pulmonary disease.

**Table 4:** Mortality from 9 March 2006 to 1 January 2015 in patients with rheumatoid arthritis who received alemtuzumab between 1991 and 1994

Age at death, years	Cause of death
78	Carcinomatosis, unknown primary, rheumatoid arthritis
79	Bronchopneumonia, rheumatoid arthritis, cellulitis
58	Respiratory failure, pulmonary oedema
89	Bronchopneumonia, congestive cardiac failure
77	Pneumonia, vascular dementia
71	Brain tumour
76	Sepsis, urinary tract infection, chronic obstructive pulmonary disease, rheumatoid arthritis
69	Adenocarcinoma of the lung
79	Pulmonary fibrosis
75	Dementia, rheumatoid arthritis

Compilation of the primary causes of death from the entire cohort from 1994 to 2015 (Table 5) showed overall 37 of the original 53 patients who received alemtuzumab had died. The predominant causes of death were cardiovascular/atherosclerotic (*n* = 12), infection (*n* = 11), and malignancy (*n* = 8). Other causes were non-malignant gastrointestinal tract perforation (*n* = 2), pulmonary fibrosis (*n* = 1), dementia (*n* = 1), upper gastrointestinal bleed (*n* = 1) and primary sclerosing cholangitis (*n* = 1).

**Table 5:** Mortality from 1994 to 1 January 2015 in patients with rheumatoid arthritis who received alemtuzumab between 1991 and 1994

	Number of patients	Primary cause of death
All deaths	37	
Cardiovascular and atherosclerotic disease	12	Ischaemic heart disease (*n* = 8), respiratory compromise secondary to heart failure (*n* = 1), multi infarct dementia (*n* = 1), bowel infarction secondary to atrial fibrillation (*n* = 1), superior mesenteric artery infarction (*n* = 1)
Infection	11	Respiratory (*n* = 10), renal tract (*n* = 1)
Malignancy	8	Lung primary (*n* = 3), non-Hodgkin’s lymphoma (*n* = 1), breast primary (*n* = 1), brain tumour (*n* = 1), stomach leiomyosarcoma (*n* = 1), unknown primary (*n* = 1)
Other	6	Non-malignant gastrointestinal tract perforation (*n* = 2), pulmonary fibrosis (*n* = 1), dementia (*n* = 1), upper gastrointestinal bleed (*n* = 1), primary sclerosing cholangitis (*n* = 1)

Morbidity in the 13 alemtuzumab patients (interview and case note review) focused on new medical diagnoses, particularly malignancy, episodes of severe infection and autoimmunity. Two patients had a new diagnosis of malignancy, both of which were skin cancers with a background of Bowen’s disease. Two had new autoimmune conditions - hyperthyroidism and coeliac disease. The remaining new diagnoses were osteoporosis, osteoarthritis, vitamin D deficiency, cardiac arrhythmia and ischaemic heart disease. There were no reports of severe infection.

### Alemtuzumab patients have persistent circulating lymphocyte abnormalities

**Fig. 1:**
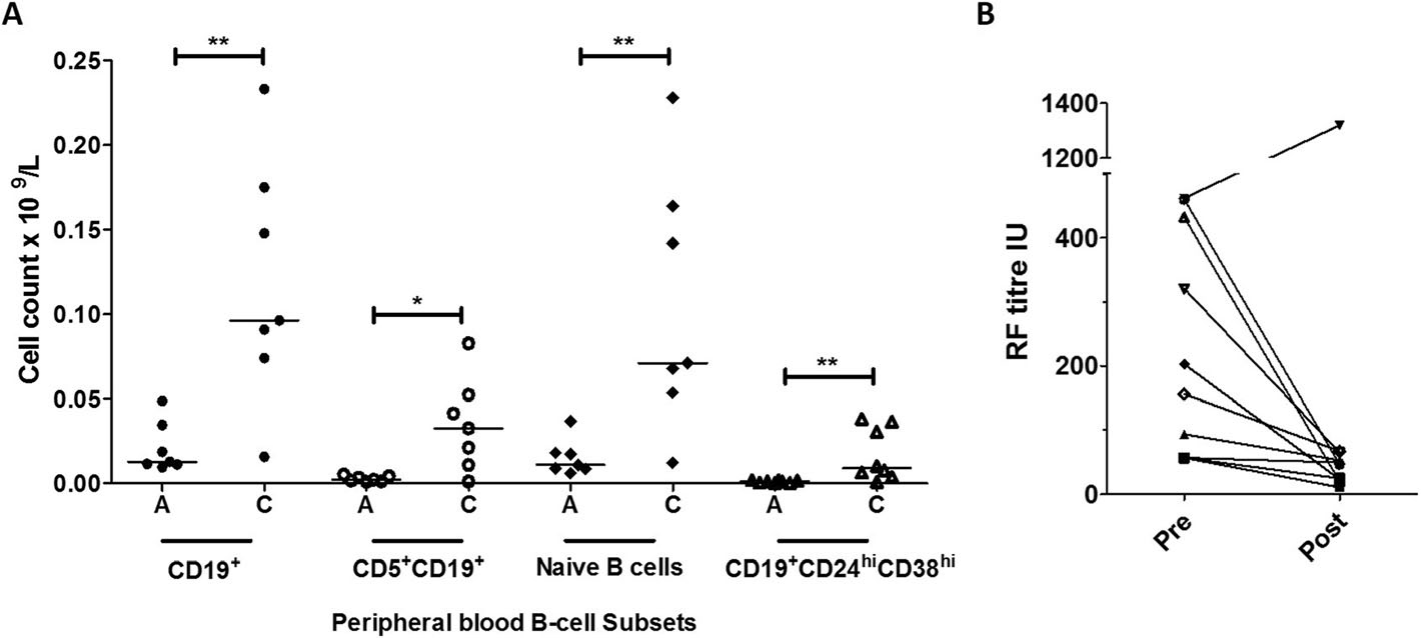
**a** Absolute counts (×10^9^/L) of different B cell populations; *A* alemtuzumab cohort, *C* controls. In the alemtuzumab cohort there was significant reduction in the frequency of CD19^+^ B cells (*p* = 0.0041), CD19^+^CD5^+^ B cells (*p* = 0.0175), naïve B cells (CD19^+^CD27^-^) (*p* = 0.0041), and CD19^+^CD24^hi^CD38^hi^ B cells (*p* = 0.003). **b** Rheumatoid factor (*RF*) titres (IU) in patients with rheumatoid arthritis (*n* = 10) pre and post treatment with alemtuzumab. The post treatment titres are either 12 years (*n* = 8) or 20 years (*n* = 2) after alemtuzumab administration. We show a clear trend in fall in RF titres in 9/10 patients but this was not statistically significant (*p* = 0.084). *p <0.05, **p < 0.01

We previously reported that at a mean follow up of 12 years after treatment, our alemtuzumab cohort remained profoundly lymphopenic [1, 3]. While our current analysis (Table 6) suggests that compared with age and sex matched disease controls, overall lymphopenia had now resolved (*p* = 0.7001), specific differences remained. CD4^+^ and CD8^+^ central memory subsets remained low (*p* = 0.0360 and *p* = 0.0274 respectively) and we noted B cell lymphopenia (*p* = 0.0041), even after excluding a patient who had recently received rituximab. This predominantly reflects reduced naïve B cells (*p* = 0.0041) but a previously highlighted deficiency of CD5^+^CD19 B cells (*p* = 0.0175) persists. There is also a significant reduction in CD19^+^CD24^hi^CD38^hi^ transitional B cells (Fig. 1a). However other previously noted abnormalities had normalised; in particular naïve and effector memory T cell populations and NK cells were not different to controls.

**Table 6:** Lymphocyte subpopulations in alemtuzumab (Alem)-treated patients and controls (CON)

Cell count, median and range (×10^9^/L)				Percentage of lymphocyte population		Previous cell count, median and range (×10^9^/L)	
Lymphocyte subpopulation	ALEM (*n* = 9*)	CON (*n* = 8)	*P* value^a^	ALEM (*n* = 9*)	CON (*n* = 8)	ALEM cohort 2012 data (*n* = 20)	ALEM cohort 2001 data (*n* = 40)
Total lymphocytes	0.93 (0.41 − 3.1)	1.125 (0.34 − 2.38)	0.7001	n/a	n/a	1.15 (0.3 − 2.9)	n/a
CD4^+^ T cells	0.37 (0.13 − 0.94)	0.48 (0.13 − 0.65)	0.4406	30.5 (11.5 − 46.7)	34.9 (13.3 − 43.7)	0.55 (0.12 − 1.94)	0.000185
CD4^+^ naïve T cells	0.23 (0.01 − 0.61)	0.21 (0.07 − 0.29)	1	20.3 (1.23 − 30.5)	13.3 (4.15 − 20.6)	0.09 (0.01 − 0.65)	n/a
CD4^+^ total memory T cells	0.13 (0.11–0.34)	0.26 (0.06 − 0.56)	0.2359	12.52 (7.26 − 18.93)	18.5 (9.3 − 29.74)	0.37 (0.10 − 0.73)	n/a
CD4^+^ central memory T cells	0.08 (0.05 − 0.16)	0.2 (0.03 − 0.42)	*0.0360*	7.24 (4.74 − 13.28)	12.82 (6.73 − 22.45)	0.11 (0.02 − 0.07)	n/a
CD4^+^ effector memory T cells	0.05 (0.02–0.17)	0.06 (0.02 − 0.14)	*0.9626*	4.37 (1.81 − 7.58)	3.78 (2.42 − 7.56)	0.26 (0.07 − 0.55)	n/a
CD8^+^ T cells	0.10 (0.04 − 0.72)	0.11 (0.05 − 0.42)	*0.9626*	7.27 (4.95 − 31.9)	9.04 (3.96 − 22.7)	0.25 (0.02 − 0.78)	0.00009
CD8^+^ naïve T cells	0.05 (0.01 − 0.14)	0.02 (0.01 − 0.08)	*0.2766*	3.32 (0.46 − 7.27)	1.57 (0.64 − 5.94)	0.05 (0.001 − 0.17)	n/a
CD8^+^ total memory T cells	0.02 (0.07 − 0.13)	0.06 (0.01 − 0.19)	*0.0592*	1.82 (0.72 − 4.41)	3.97 (0.62 − 0.55)	0.12 (0.01 − 0.41)	n/a
CD8^+^ central memory T cells	0.01 (0.003 − 0.01)	0.02 (0.005 − 0.11)	*0.0274*	0.69 (0.26 − 1.33)	1.84 (0.34 − 6.01)	0.02 (0.003 − 0.12)	n/a
CD8^+^ effector memory T cells	0.01 (0.007–0.07)	0.03 (0.007 − 0.1)	*0.1672*	1.51 (0.45 − 3.2)	1.94 (0.31 − 5.72)	0.07 (0.01 − 0.37)	n/a
B cells	0.01 (0.01 − 0.05)	0.09 (0.02 − 0.2)	*0.0041*	1.7 (1.12 − 4.15)	8.96 (4.64 − 17.5)	0.08 (0.02 − 0.26)	0.000115
Naïve B cells	0.01 (0.001–0.02)	0.08 (0.7 − 0.14)	*0.0041*	1.18 (0.71 − 2.69)	5.47 (3.62 − 16.4)	0.06 (0.01 − 0.23)	n/a
Memory B cells	0.006 (0.002 − 0.02)	0.01 (0.008–0.03)	*0.1282*	0.56 (0.37 − 0.86)	1.19 (0.22 − 3.91)	0.02 (0.002 − 0.19)	n/a
CD5^+^ B cells	0.001 (0.001 − 0.005)	0.03 (0.001 − 0.08)	*0.0175*	0.28 (0.084 − 0.94)	2.22 (0.33 − 3.48)	0.005 (0.0009 − 0.03)	n/a
CD19^+^CD24^hi^CD38^hi^ B cells	0.001 (0.00005 − 0.002)	0.009 (0.0008 − 0.038)	*0.003*	0.061 (0.014 − 0.42)	0.70 (0.51 − 2.31)	n/a	n/a
NK cells	0.1 (0.02 − 0.17)	0.07 (0.02 − 0.10)	0.4234	10.5 (3.3 − 19.2)	8.35 (4.84 − 13.3)	0.06 (0.01 − 0.2)	n/a
NK T cells	0.01 (0.0006–0.13)	0.009 (0.002 − 0.02)	0.6730	0.76 (0.12 − 4.34)	0.73 (0.52 − 1.27)	0.05 (0.003–0.27)	n/a

Median values and ranges are displayed for both absolute number and percentage of lymphocyte population. ^a^ *P* value for alemtuzumab versus established rheumatoid arthritis cell count absolute number); values in *italics* are significant. Gating strategies were as follows: CD4^+^ T cells: CD3^+^CD4^+^; CD4^+^ naïve T cells: CD3^+^CD4^+^CD45RA^+^CD62L^+^; CD4^+^ total memory T cells: CD3^+^CD4^+^CD45RA^-^; CD4^+^ central memory T cells: CD3^+^CD4^+^CD45RA^-^CD62L^+^; CD4^+^ effector memory T cells: CD3^+^CD4^+^CD45RA^-^CD62L^-^; CD8^+^ T cells: CD3^+^CD4^-^, CD8^+^ naïve T cells: CD3^+^CD4^-^CD45RA^+^CD62L^+^; CD8^+^ total memory T cells: CD3^+^CD4^-^CD45RA^-^; CD8^+^ central memory T cells: CD3^+^CD4^-^CD45RA^-^CD62L^+^; CD4^+^ effector memory T cells: CD3^+^CD4^-^CD45RA^-^CD62L^-^; B cells: CD19^+^; Naïve B cells: CD19^+^CD27^-^; Memory B cells: CD19^+^CD27^+^; CD5^+^ B cells: CD19^+^CD5^+^; CD19^+^CD24^hi^CD38^hi^ B cells: CD19^+^CD24^hi^CD38^hi^; NK Cells: CD3^-^CD56^+^, NK T Cells: CD3^+^CD56^+^. For comparison previous analyses (years 2001 and 2012) of lymphocyte subpopulations of the alemtuzumab cohort are included. *Single patient had rituximab within the preceding month and was excluded from B-cell analyses. *NK* natural killer. *n/a* not available

### Seropositive patients with RA have persistently reduced RF titres following alemtuzumab therapy

We reviewed RF titres as documented in the medical notes at the time of alemtuzumab administration. Of our current alemtuzumab cohort only two patients had positive RF at the time of alemtuzumab therapy, but these values were now reduced compared to baseline values 20 years earlier (432 → 20 IU/ml and 57 → 25 IU/ml). We therefore looked at data from eight additional (deceased) RF-positive patients who had received alemtuzumab, and compared their baseline RF titres with titres published at their last follow up [2]. Whilst not being statistically significant (*p* = 0.084), there was a reduction in RF titres 12 years after alemtuzumab treatment (Fig. 1b).

### Vaccine responses in patients receiving alemtuzumab and in controls

All patients who attended interview were offered vaccination, dependent on their vaccination status at the time. Four patients on alemtuzumab and three controls received influenza vaccine, the remainder having already received seasonal influenza vaccine prior to recruitment. For these latter patients we assessed the seroprotection rate only. Seven patients on alemtuzumab and six controls received pneumococcus vaccination, the others having been vaccinated within the last 5 years (*n* = 2) or having declined vaccination (*n* = 2). Six patients on alemtuzumab and four control patients received the combined diphtheria, tetanus and polio vaccine. There were no significant adverse events following any vaccination.

Due to the small numbers in both groups, robust statistical comparison was not possible. Nonetheless we observed similar levels of seroprotection following poliovirus (P1-P3), tetanus and diphtheria vaccination, whereas for pneumococcal antigen, seroprotection appeared higher in the alemtuzumab cohort. Seroconversion was comparable for tetanus and diphtheria, although potentially lower in alemtuzumab recipients for polio. For influenza seroprotection, the rates in the 4 weeks post vaccine were similar in both cohorts, although the seroconversion rate was uniformly poor. When including those who had influenza vaccines administered outside this study we saw a lower seroprotection rate in the alemtuzumab patients; however, the timing of the prior vaccination was unknown, making meaningful comparison difficult (Table 7).

**Table 7:** Responses to vaccination in alemtuzumab (ALEM) and control cohorts (CON)against diphtheria, tetanus, polio virus (P1-P3), pneumococcal antigen and influenza

	Diphtheria toxoid		Tetanus Toxoid		Poliovirus							
					P1		P2		P3		Pneumococcus antigen	
	Alem (*n* = 6)	Controls (*n* = 4)	Alem (*n* = 6)	Con (*n* = 4)	Alem (*n* = 6)	Con (*n* = 4)	Alem (*n* = 6)	Con (*n* = 4)	Alem (*n* = 6)	Con (*n* = 4)	Alem (*n* = 7)	Con (*n* = 6)
Seroprotection rate, %	66	25	100	100	100	100	83.3	100	83.3	100	_	
Seroconversion rate, %	33	25	66.7	50	50	100	16.6	100	83.3	100		
% Satisfactory response	_										66	0
Influenza vaccine titres			A/Cal/7/09		A/Texas/50/12		B/Mass/02/12					
			Alem	Con	Alem	Con	Alem	Con				
Patients vaccinated at interview Alem *n* = 4 Controls, *n* = 3	GMT pre-vaccination		2.92	4.82	3.27	5.96	1.88	2.74				
	GMT post-vaccination		3.42	5.16	3.53	6.17	1.98	2.61				
	Seroconversion factor		1.16	1.07	1.08	1.03	1.05	0.85				
	Seroprotection rate, %		50	66.7	50	100	25	33.3				
	Seroconversion rate, %		25	0	0	0	0	0				
Alem* *n* = 9 Controls* *n* = 8	Seroprotection rate, %		22	87.5	56	87.5	11	37.5				

Tetanus and diphtheria seroprotection was achieved when respective IgG titres were >1.0 IU/ml and seroconversion was defined as new seroprotection. For polio subtypes (P1-P3) seroprotection was a neutralizing antibody titre of ≥1:8 and seroconversion a ≥ fourfold increase in titres. Satisfactory response for pneumococcal antigen was defined as a twofold or more increase from vaccination baseline in antibody concentrations in six or more of 12 pneumococcal serotypes (1, 3, 4, 5, 6B, 7 F, 9 V, 14, 19A, 19 F, 23 F and 18C). For influenza haemagglutination inhibition (HAI) titres >1:40 were defined as seroprotective and seroconversion was defined as rises from negative titres to values of =/>1:40 (or fourfold titre increase if values were above baseline). Seroconversion factor was defined as the fold increase in geometric mean HAI titres (GMT) post-vaccination (recommended ≥2) and seroconversion rate the percentage of vaccines with an increase in HAI titre ≥ fourfold following vaccination (recommended >30%). Seroprotection rate (for all) was defined as percentage of group achieving seroprotection. *Including patients who had influenza vaccine out with this study period

### Cytokines

**Fig. 2:**
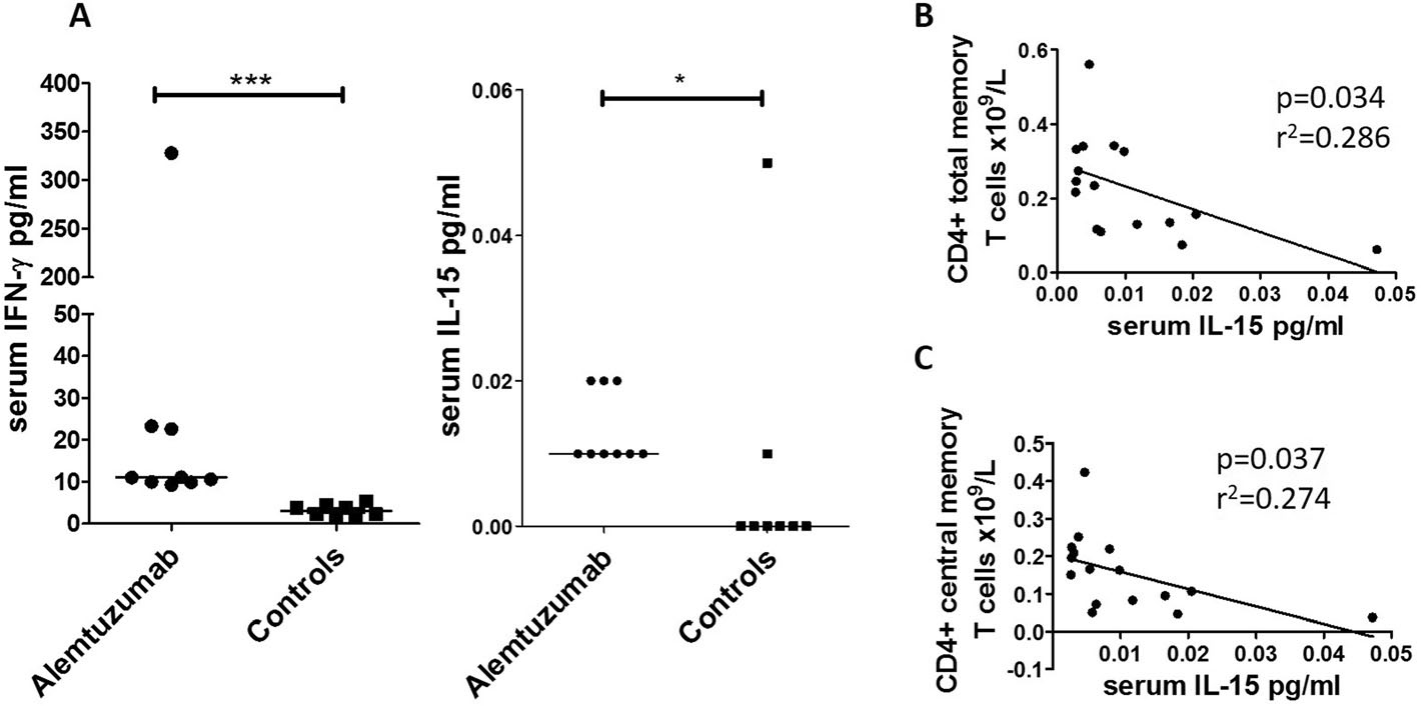
**a** Circulating levels of serum IFN-γ and IL-15 (pg/ml) in the alemtuzumab cohort and controls. We show a significant increase in IFN-γ (*p* < 0.0001) and IL-15 (*p* = 0.0188) in the alemtuzumab cohort. **b**, **c** CD4^+^ total memory and central memory T cells (cell number × 10^9^/L) demonstrated significant inverse associations with serum IL-15 (pg/ml) (*p* = 0.034, *r* ^2^ = 0.286 and *p* = 0.037, *r* ^2^ = 0.274 respectively). Results were considered significant when the *p* value was <0.05. *p < 0.05, ***p < 0.0001

There were significantly higher serum IFN-γ (*p* < 0.0001) and IL-15 (*p* = 0.019) levels detected in the alemtuzumab cohort compared with controls (Fig. 2a). There was a significant inverse association between serum IL-15 and CD4^+^ total memory and central memory T cells (*p* = 0.034 and *p* = 0.037), see Fig. 2b and c. A similar trend for CD8^+^ total memory and central memory T cells was also seen albeit not significant (*p* > 0.05). There were no significant associations detected in the effector memory compartments.

## Discussion

Lymphocyte depleting therapies continue to be used in autoimmunity and transplantation [4, 6]; indeed, in a current phase-I clinical trial investigating the therapeutic potential of stem cell transplantation for RA, alemtuzumab is being administered as part of the immunosuppressive regimen [7]. Furthermore, novel targets of immunomodulation such as the JAK inhibitor tofacitinib can also reduce CD4^+^ and CD8^+^ T cells and natural killer (NK) cells in vivo [8]. Thus, immune reconstitution is of relevance to a growing range of therapies. Here we present 20-year follow-up data on a unique cohort of patients with RA treated with the lymphodepleting agent alemtuzumab.

Causes of death in this most recent follow up are in keeping with our previous observations, with four cases of infection (three pulmonary), three tumours, and single cases of respiratory failure (secondary to pulmonary oedema), dementia and pulmonary fibrosis (Tables 4 and 5). We previously demonstrated that overall mortality did not differ between alemtuzumab recipients and a comparable (external) control population. However, the original control cohort is no longer under follow up, and cause-specific mortality comparisons were not performed due to the small size of the alemtuzumab cohort. Overall, however, the major causes of death (cardiovascular, pulmonary infection and malignancy, particularly pulmonary) is in keeping with other established RA cohorts [9].

We documented two new cases of autoimmunity (hyperthyroidism and coeliac disease) but secondary autoimmunity has been much less common in RA recipients of alemtuzumab than in multiple sclerosis (MS) cohorts. In the latter, autoimmunity affected approximately one third of alemtuzumab recipients and peaked after 2 years. It is mainly, but not exclusively, thyroid in nature and has been linked to tolerance breakdown during the homeostatic proliferation component of immune reconstitution [10]. Additionally a recent retrospective study of alemtuzumab use in Bechet’s disease demonstrated that approximately two thirds of patients achieved lymphocyte recovery by a median of 9 months, but 25% of patients developed secondary autoimmune thyroid disease [11]. Whilst patients affected by these diseases clearly inherit distinct genetic backgrounds [12], our data demonstrate that patients with RA reconstitute more slowly than their counterparts with MS or indeed Bechet’s, which may reduce the incidence of secondary autoimmunity.

Total lymphocyte counts in our alemtuzumab cohort were now comparable with age, sex and disease duration-matched RA controls but significant reductions in some lymphocyte subsets remained. These persistently deranged lymphocyte subsets are remarkable as alemtuzumab does not target haemopoietic precursors and its half-life, when used in the context of autoimmunity, is up to 5–6 days, depending on the dosing regimen [13, 14]. There is a reduction in CD19^+^ B cells, comprising a persistent reduction in CD5^+^ B cells, and a newly identified reduction in naïve B cells. CD5^+^ B cells have previously been reported to be elevated in autoimmune conditions and involved in autoantibody production, including RF [15–17]. Interestingly we also noted a fall in RF titres following alemtuzumab therapy in concert with the reduction in CD5^+^ B cells. A potential caveat of this observation relates to the technical differences in RF measurement over the intervening years, thus, confounding these findings. Nonetheless, in the 1970s the World Health Organisation proposed the use of international RF measurement units [18], which markedly reduced inter-lab variability. Whilst not measured at baseline, anti-CCP titres were much lower in the alemtuzumab cohort than in matched controls (Tables 1 and 2). More recently CD5^+^ B cells have been reported to have a regulatory function [19–21], so we also examined the frequency of CD19^+^CD24^hi^CD38^hi^ transitional B cells, which have putative regulatory function and are known to be reduced in RA [21, 22]. This subset was further reduced in the alemtuzumab cohort compared with the control cohort.

Despite reduced B cell numbers, and small numbers of patients receiving vaccines, the vaccine responses appeared comparable in the alemtuzumab and control cohorts. Overall responses were generally poorer than previously published in RA but both cohorts had substantially longer disease duration than those included in previously published work [23]. None of the vaccines administered were true neo-antigens but we concluded that immune memory appears largely maintained in the long term following alemtuzumab treatment.

A further unexpected finding was elevated levels of circulating IL-15 and IFN-γ in our alemtuzumab cohort. IL-15 closely resembles IL-2 in tertiary structure and is important for T cell homeostasis. Central memory CD4^+^ and CD8^+^ T cells remained significantly reduced in the current analysis and we observed a significant inverse association between IL-15 and central memory CD4^+^ T cells and a similar trend in central memory CD8^+^ T cells. IL-15 enhances the function and inhibits apoptosis of human CD4^+^ and CD8^+^ effector memory cells, which is in keeping with our observed normal effector memory CD8^+^ and CD4^+^ cell frequency [24]. In addition effector memory CD4^+^ T cells are relatively resistant to alemtuzumab, which may have contributed to these observations [25, 26]. IL-15 also has potent effects on NK cells, which proliferate and secrete IFN-γ [27, 28]. We have previously emphasised the lack of serious and opportunistic infections in lymphopenic alemtuzumab recipients, which could be underpinned by such homeostatic mechanisms.

## Conclusions

In conclusion, 20–25 years after initial administration of alemtuzumab, causes of death remained consistent with those associated with long-standing RA. We continued to see significant perturbations in lymphocyte subsets, noting for the first time a reduction in naïve B cells and CD19^+^CD24^hi^CD38^hi^ potential Bregs, and a persistent reduction in CD5^+^ B cells. Nonetheless, limited data suggest that vaccine responses reflecting immune memory are maintained. We also documented, for the first time, elevated circulating IL-15 and IFN-γ levels. Our data are reassuring and provide a unique insight into the long-term consequences of potent lymphodepleting therapy in patients with RA. Whilst alemtuzumab is no longer used in this setting, our findings are relevant to its use in other settings and to other current and future targeted therapies, and emphasise the importance of long-term follow up.

## Abbreviations

ABA: abatacept
ADA: adalimumab
anti-CCP antibody: anti-cyclic citrullinated peptide antibody
CRP: C-reactive protein
DAS-28: disease activity score-28
DMARD: disease modifying anti-rheumatic drug
ESR: erythrocyte sedimentation rate
ETA: etanercept
GM-CSF: granulocyte macrophage colony stimulating factor
GMT: geometric mean titres
HAI: haemagglutination inhibition
HAQ: health assessment questionnaire
HQ: hydroxychloroquine
IFN-γ: interferon-γ
Ig: immunoglobulin
IL: interleukin
LEF: leflunomide
MS: multiple sclerosis
MTX: methotrexate
NK cells: natural killer cells
RA: rheumatoid arthritis
RF: rheumatoid factor
SJC: swollen joint count
SSZ: sulphasalazine
TJC: tender joint count
TNF-α: tumour necrosis factor α
TOC: tocilizumab
VAS: visual analogue scale
